# Sex difference in effect of ankle landing biomechanics in sagittal plane on knee valgus moment during single-leg landing

**DOI:** 10.1038/s41598-022-23675-y

**Published:** 2022-11-05

**Authors:** Jinkyu Lee, Choongsoo S. Shin

**Affiliations:** Department of Mechanical Engineering, Sogang University, 35 Baekbeom-Ro, Mapo-Gu, Seoul, 04107 Republic of Korea

**Keywords:** Human behaviour, Risk factors

## Abstract

Ankle landing strategies affects the biomechanical characteristics of the knee joint, especially knee frontal plane loading. However, no studies have investigated whether the association between ankle landing biomechanics in sagittal plane and the knee frontal plane loading differs between sexes. The purpose of this study was to examine whether there is a sex difference in the effect of ankle plantar flexion at the contact angle, ankle range of motion (ROM), and ankle plantar flexion moment on knee valgus loading during single-leg landing. Twenty-five females and twenty-four males performed a single-leg landing. Joint kinematics and kinetics of the lower extremities were measured. The relationship between ankle biomechanics in the sagittal plane (ankle plantar flexion angle at contact, ROM, and peak ankle plantar flexion moment) and peak knee valgus moment were analyzed. In males, the larger ankle plantarflexion angle at contact and ROM were significantly associated with lower peak knee valgus moment. In addition, in males only, a greater peak ankle plantar flexion moment was significantly associated with a lower peak knee valgus moment and greater peak ankle inversion moment. Altering ankle landing strategies in the sagittal plane during single-leg landing may reduce the knee valgus moment, which is one of risk factors for anterior cruciate ligament injury, in males only.

## Introduction

Non-contact anterior cruciate ligament (ACL) injury in females occurs at a higher rate than in males during sports activities^1^. One possible mechanism underlying the ACL injury rate difference is biomechanical or neuromuscular factors, such as excessive knee valgus loading (angle and moment) and/or muscle imbalance^2,3^. In particular, females have experienced more ACL injuries related to knee valgus loading than males^4^. Although knee valgus loading can be generated by ankle, knee, and hip motions and moments^5–7^, most ACL injury-related studies have only focused on the knee and hip, and less attention has been paid to the ankle joint.

Ankle frontal plane motion is linked to frontal plane motion of the knee^8^. Observational study has suggested that ankle eversion with hip adduction creates dynamic knee valgus^9^. During landing, the ankle plantar-flexor muscles absorb the landing impact. The gastrocnemius and soleus muscles mainly produce ankle plantar flexion, and the tibialis posterior muscle supports ankle plantar flexion while producing ankle inversion. Thus, plantar flexion is usually coupled with inversion^10^. In addition, Vieira et al. reported that plantar flexor (medial gastrocnemius muscle) contraction in a static position exerts an ankle inversion moment with plantarflexion moment, suggesting a positive linear relationship between ankle plantar flexion moment and ankle inversion moment^11^. This coupling of ankle plantar flexion and inversion suggests that alterations in the sagittal plane of the ankle biomechanics during landing may affect the frontal plane of the knee joint. This hypothesis was reasonably supported by findings from previous study^12^. Recently, Lee and Shin reported that the initial contact ankle angle in the sagittal plane can reduce the peak knee valgus moment in male subjects^12^. Therefore, ankle control in the sagittal plane is likely to be one of the factors causing differences in knee valgus loading during landing in only male. However, to best of our knowledge, there is no studies regarding the relationship between ankle control in the sagittal plane and knee valgus moment in females and whether the sex difference exists in that relationship.

Several studies have reported conflicting results regarding sex differences in ankle landing strategies. Decker et al. suggested that females dissipate the landing impact with more plantar flexion of the ankle joint at initial contact, thereby obtaining a larger ankle range of motion (ROM)^13^. Kernozek et al. reported that although there was no sex difference in ankle plantar flexion angle at initial contact, females exhibited larger ankle ROM owing to a greater maximum ankle dorsiflexion angle^14^. In contrast, Ali et al. showed that males predominantly landed with plantar flexion and females landed in a relatively dorsiflexed position^15^. Whether there are differences in ankle landing strategies between males and females is debatable, but it is commonly suggested that ankle landing strategies in the sagittal plane can influence shock attenuation. Lee et al. (2018) reported that the absorption in the ankle joint and entire lower extremity joint, measured as a joint negative work, are positively correlated with the ankle plantar flexion angle at initial contact^16^. An ankle landing strategy incorporating a greater ankle ROM owing to the increase in ankle plantar flexion angle at initial contact affords greater shock attenuation, resulting in an increased plantar flexor moment during landing^6,17^. As the ankle plantarflexion angle at initial contact increased, the ankle contribution to the lower extremity joint moment increased, and the peak landing force and loading rate decreased^18^.

The general consensus of these studies suggests that a greater shock attenuation at the ankle joint, such as a large ankle ROM (owing to great ankle plantar flexion at initial contact) or a greater ankle plantar flexion (flexor) moment during landing, may protect the ACL. However, it remains unknown how this greater attenuation at the ankle joint affects knee valgus loading, which can lead to an ACL injury in a sex-dependent manner. Thus, the purpose of the current study was to determine whether there is a sex difference in the effect of ankle plantar flexion at the initial contact angle, ROM, and plantar flexion moment on knee valgus loading during single-leg landing. It was hypothesized that the relationship between ankle plantarflexion angle at initial contact and peak knee valgus moment, and peak ankle plantar-flexion moment and peak knee valgus moment would differ between sexes.

## Results

### Sex difference

Females showed significantly greater ankle plantar flexion angles at initial contact and ROM during single-leg landing (*p* < 0.001, *d* = 1.08 and *p* < 0.001, *d* = 1.10, respectively, Table 1). There were no significant differences between the sexes in the knee flexion angle at initial contact and ROM. Females had significantly less peak vGRF (*p* = 0.004, *d* = 0.79, Table 1) and delayed time to peak vGRF (*p* = 0.033, *d* = 0.63, Table 1) than males. There were no significant differences between the sexes in peak ankle plantar flexion (*p* = 0.765, *d* = 0.09), inversion moment (*p* = 0.110, *d* = 0.46), knee extension (*p* = 0.521, d = 0.18), and valgus moment (*p* = 0.483, *d* = 0.20, Table 1).

**Table 1:** The mean (standard deviation) value of sex comparison of biomechanical variables during single-leg landing.

Variable	Male	Female	*P* value	Cohen’s *d*
**Ankle plantarflexion angle at initial contact (degree)**	**27.7 (6.6)**	**34.1 (5.2)**	**< 0.001**	**1.08**
Initial contact knee flexion angle (degree)	4.1 (3.5)	6.4 (4.8)	0.057	0.56
Maximum ankle dorsiflexion angle (degree)	14.9 (4.4)	16.5 (4.8)	0.228	0.35
**Maximum knee flexion angle (degree)**	**43.2 (5.8)**	**47.4 (6.6)**	**0.023**	**0.67**
**Ankle ROM in sagittal plane (degree)**	**42.6 (8.8)**	**50.6 (5.5)**	**< 0.001**	**1.10**
Knee ROM in sagittal plane (degree)	39.2 (4.4)	41.0 (4.1)	0.136	0.43
**Time to peak vertical GRF (ms)**	**58.3 (11.6)**	**64.4 (6.8)**	**0.033**	**0.63**
**Peak vertical GRF (N/BW)**	**2.7 (0.5)**	**2.4 (0.2)**	**0.004**	**0.79**
Peak ankle plantarflexion moment (N/(BW*Ht))	0.14 (0.04)	0.14 (0.02)	0.765	0.09
Peak ankle inversion moment (N/(BW*Ht))	0.03 (0.03)	0.02 (0.02)	0.110	0.46
Peak knee extension moment (N/(BW*Ht))	0.18 (0.04)	0.17 (0.03)	0.521	0.18
Peak knee valgus moment (N/(BW*Ht))	0.07 (0.02)	0.08 (0.02)	0.483	0.20

Significant values are in bold.

### Correlation between ankle kinematics in sagittal plane and peak knee valgus moment

**Figure 1:**
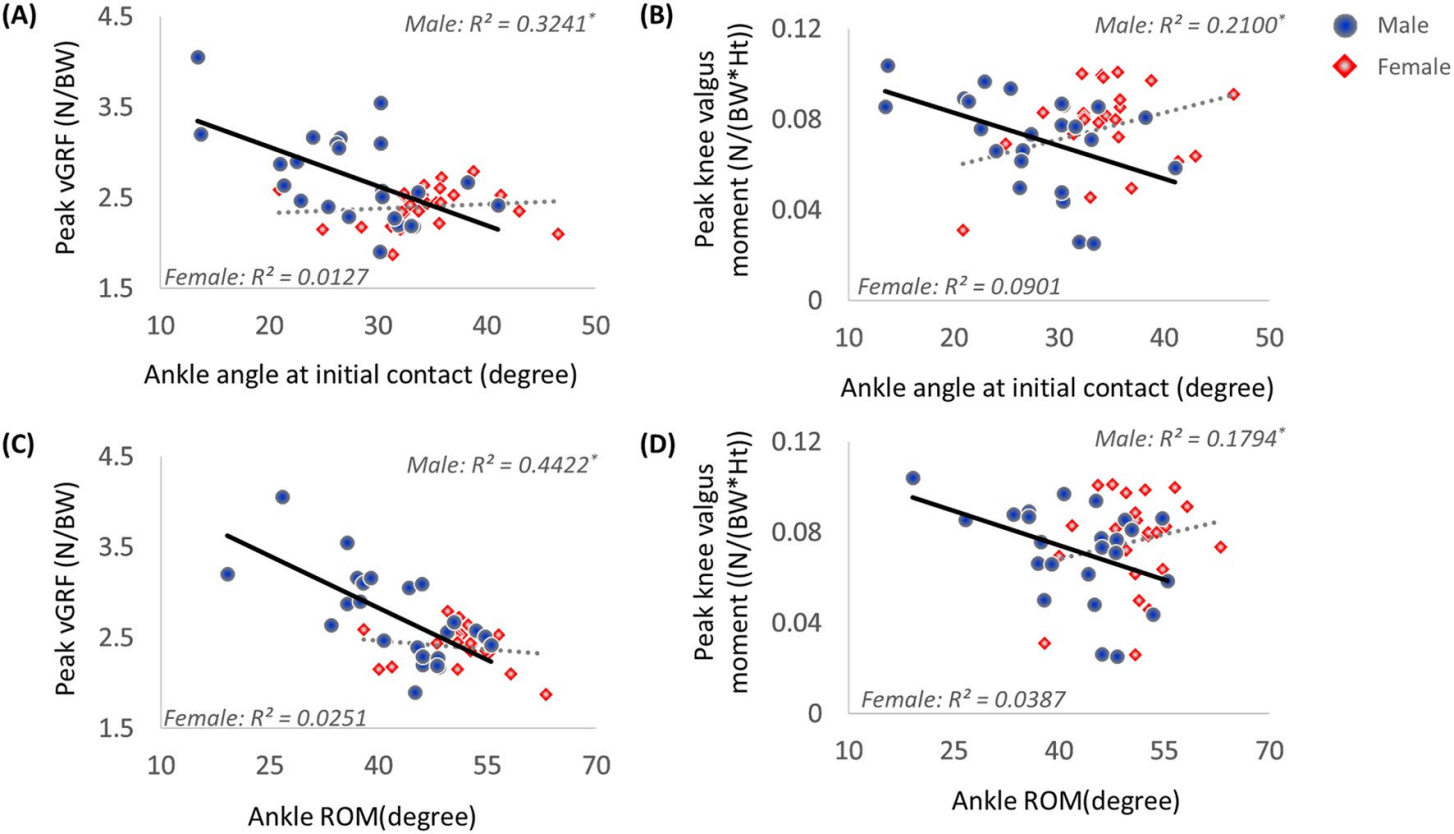
Relationships between peak vGRF and knee valgus moment and ankle kinematics in sagittal plane. Scatter plots depicting the relationship between ankle plantarflexion angle at initial contact and (**A**) peak vGRF and (**B**) knee valgus moment. Scatter plots depicting relationship between ankle ROM in sagittal plane and (**C**) peak vGRF and (**D**) knee valgus moment.

The peak vGRF and knee valgus moment in males showed a significant negative correlation with ankle plantar flexion angle at initial contact (*r* =  − 0.569, *p* = 0.036) and ROM (*r* =  − 0.665, *p* < 0.001) (Fig. 1). In females, the peak vGRF and knee valgus moment were not significantly correlated with ankle plantar flexion angle at initial contact (*r* = 0.113, *p* = 0.592 and *r* = 0.300, *p* = 0.145, respectively) and ROM (*r* = − 0.156, *p* = 0.450 and *r* = 0.197, *p* = 0.346, respectively).

### Correlation between peak ankle plantarflexion moment and peak knee valgus moment

**Figure 2:**
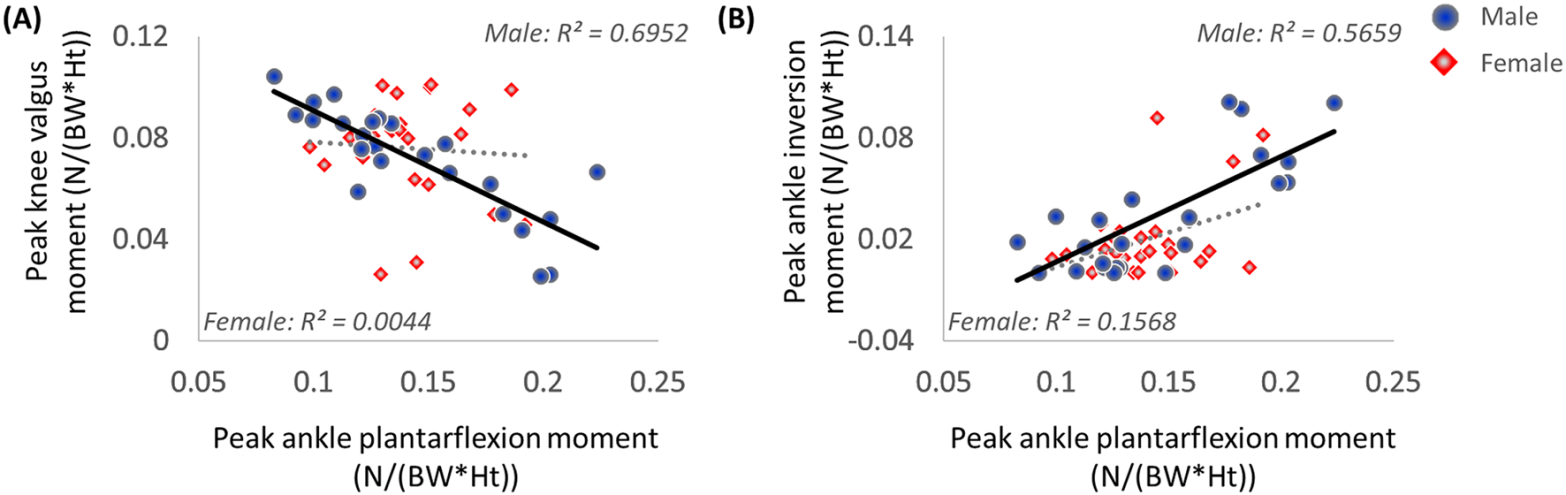
Scatter plots depicting the relationship between: (**A**) peak ankle plantarflexion and peak knee valgus moments and (**B**) peak ankle plantarflexion and inversion moments.

Only males had a significant negative correlation between the peak ankle plantarflexion and the knee valgus moment (*r* = – 0.834, *p* < 0.001) (Fig. 2A). In females, there was no significant correlation between the peak ankle plantar flexion and the knee valgus moment (*r* = − 0.066, *p* = 0.751).

### Correlation between peak ankle plantarflexion moment and peak ankle inversion moment

The peak ankle plantarflexion moment in males was significantly positively correlated with the peak ankle inversion moment (*r* = 0.752, *p* < 0.001) (Fig. 2B). In females, there was no significant correlation between the peak ankle plantar flexion and the inversion moment (*r* = 0.396, *p* = 0.051).

## Discussion

Differences in knee valgus loading between sexes have been reported in previous studies focusing on knee joint motion and related muscles during landing^19–21^. However, recent studies suggest that ankle-to-knee biomechanical interaction may influence knee frontal plane motion^12,22,23^. Thus, the purpose of this study was to determine whether sex differences exist in the association between sagittal ankle control (ankle initial contact angle (ankle ROM) and plantar flexion moment) and knee valgus moment. An analysis of the correlation between sagittal ankle control and the knee valgus moment was performed for both males and females. Only the male subjects in this study showed a significant correlation between ankle sagittal kinematics/kinetics and the knee valgus moment. Although the peak knee valgus was not significantly different between sexes, the findings in this study support our hypothesis.

Regarding the male subjects, it was found that the peak vGRF and peak knee valgus moment were negatively correlated with the ankle plantar flexion angle at initial contact and ROM. This result suggests that in the case of males, it is important to use a large ankle plantarflexion angle at initial contact and/or ROM to effectively absorb the landing impact and reduce the knee valgus loading. Our results agree with previous findings that landing with a large ankle plantar flexion angle at initial contact or ROM reduces landing impact^15,16,18^. In addition, the GRF during landing can induce frontal plane loading in the lower extremity joint^24,25^. Moreover, Hewett et al. reported that the peak knee valgus moment in females was more positively correlated with the peak landing force compared with flexion and extension moments and valgus moment at the knee, hip, and ankle during landing^26^. In this study, females showed a larger plantarflexion angle at initial contact and ROM than males, but there was no significant correlation with peak vGRF. This may imply that landing with a large ankle plantarflexion angle at initial contact or ROM may have played a more important role in reducing the knee valgus loading in males than in females through landing force attenuation. The reason why only males showed a significant relationship cannot be determined through this study, and further study on the mechanism of *sex* differences in this relationship is warranted.

Our results indicate that more ankle plantar flexion moment was associated with more ankle inversion moment and less knee valgus moment in males, but not in females. One might suppose from these results that females may not be able to generate sufficient ankle inversion moment coupled with plantarflexion moment to counteract knee valgus. Ankle plantar-flexor muscles (gastrocnemius, soleus, and tibialis posterior) act more dominantly during landing than dorsiflexors (tibialis anterior)^27^, and previous studies^11,28^ have suggested that the triceps muscle force produces the ankle inversion moment in the sitting position. In addition, the tibialis posterior and anterior muscles function as invertors of the foot^29^. Thus, ankle muscles related to plantar flexion and dorsiflexion may be accompanied by ankle inversion during landing. With regard to the ankle frontal plane moment at landing, Thompson et al.^30^ suggested the possibility of an association between the ankle frontal plane moment and the knee frontal plane moment during double-leg landing. Thompson et al.^30^ reported that the intervention group who participated in an ACL injury prevention program showed increased peak ankle eversion moments and peak knee valgus moments during double-leg landing. Excessive ankle eversion has been observed with dynamic knee valgus when non-contact ACL injury occur^6^. Joseph et al.^31^ suggested that restricting ankle eversion reduces knee valgus motion. Although there was no significant difference in the peak ankle inversion moment between sexes, only males in this study showed a significant correlation between ankle peak plantar flexion and the inversion moment. This result suggests that sex differences in ACL injury risk related to knee valgus loading may be partially explained by differences in coupling between ankle plantarflexion and the inversion moment.

In addition, the positive relationship between ankle plantarflexion moment and the inversion moment, which exists only in males, may suggest that there is a sex difference in the dynamic balance of the entire body during single-leg landing. The ankle plantar flexor muscles play an important role in controlling postural stability during single-leg standing^32^. Vieira et al.^11^ reported that the peak ankle inversion moment amounted to approximately 13% of the peak ankle plantarflexion moment in the sitting position and suggested that these relationships between ankle plantarflexion and the inversion moment play a role in the mediolateral balance of the entire body. Activation of the ankle muscle (gastrocnemius muscle) with other shank muscles provides stability to the body during single-leg standing^33^. It is relatively easier to lose body balance with a single-leg landing than with a double-leg landing because of the shift of the center of mass of the entire body^34^; thus, only individual landing legs need to absorb the landing impact by the muscle–tendon unit^35^. A video analysis study demonstrated that the loss of body balance during single-leg landing is frequently observed at the incidence of an ACL injury^36^. In this study, the peak ankle inversion moment amounted to approximately 21% of the peak ankle plantar flexion moment for males during single-leg landing. Collectively, the greater ankle plantarflexion moment with the inversion moment may reduce the risk of ACL injury by improving balance during single-leg landing in males.

In conclusion, the effects of ankle plantar flexion angle at initial contact, ROM, and ankle plantar flexion moment on the knee valgus moment differed between sexes during single-leg landing. Only males demonstrated that peak knee valgus moment was negatively correlated with the peak ankle plantar flexion moment, ankle plantar flexion angle at initial contact, and ROM. In addition, in males only, the peak ankle plantar flexion moment was positively correlated with the peak ankle inversion moment. These findings suggest that altering ankle landing strategies in the sagittal plane during single-leg landing may reduce the knee valgus moment, which is one of risk factors for ACL injuries, in males only. The current investigation may help explain one of the reasons underlying the phenomenon of sex differences in ACL injury related to the knee valgus moment.

## Method

Forty-nine recreationally active participants, including 25 females (age: 21.2 ± 1.3 years, weight: 54.7 ± 4.5 kg, height: 1.64 ± 0.03 m) and 24 males (age: 24.0 ± 4.2 years, weight: 72.6 ± 8.5 kg, height: 1.75 ± 0.04 m) were recruited. Participants were excluded if they had a lower extremity injury experienced in the past 6 months that prevented participation in physical activity for more than 2 weeks, or a history of ACL injuries or other musculoskeletal injuries requiring surgery. Although no study has examined the relationship between ankle kinematics and knee valgus moment in females, the correlation coefficient in males between ankle plantar-flexion angle at initial contact and peak knee valgus was reported to be a 0.5 in the previous study^12^. Therefore, the same correlation coefficient of 0.5 for the females was assumed in this study. To determine the relationship between ankle plantar-flexion angle at initial contact and peak knee valgus between the sexes, the necessary minimum number of participants for male and female was 23, as calculated from a statistical power of 0.8 with an alpha level of 0.05 and a correlation coefficient of 0.5 using G*power (version 3.1.9.7, Heinrich-Heine-Universität Düsseldorf, Germany).

This study was approved by the ethics committee of the Sogang University (SGUIRB-A-1808-44). Prior to participation, all participants signed an informed consent form. All methods in this study were conducted according to the relevant guidelines and regulation of the Declaration of Helsinki.

**Figure 3:**
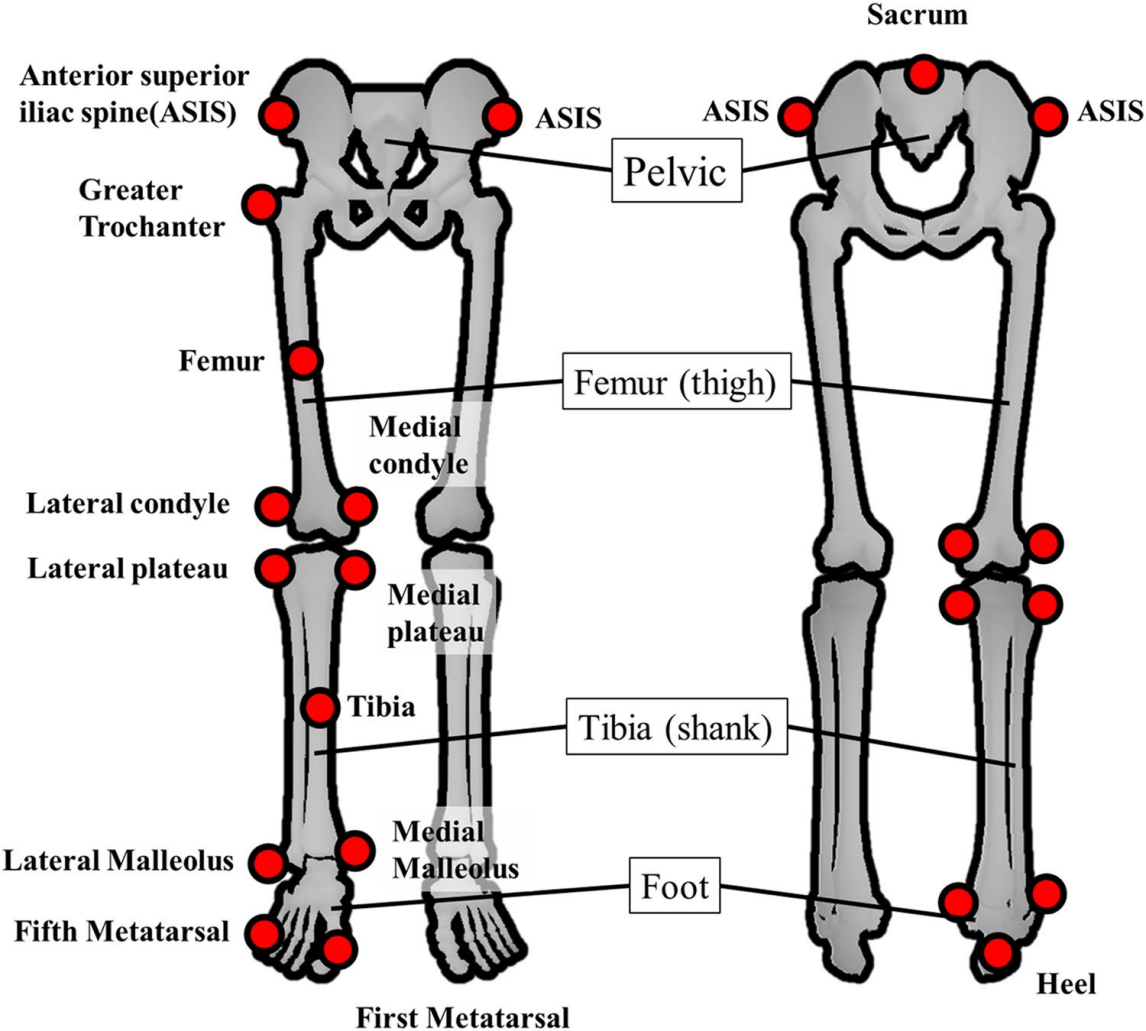
Lower extremity markers configuration: anatomical site of the marker placement.

The ground reaction force (GRF) data were recorded using a floor-embedded force plate (9260AA6; Kistler, Winterthur, Switzerland) at a rate of 1200 Hz. A motion capture system equipped with 8 infrared cameras (7 Eagle and 1 Raptor; Motion Analysis Corp., Santa Rosa, CA, USA) recorded the 3D coordinates of 15 precisely attached external reflective markers located on the dominant leg at the following locations^16^: bilateral anterior superior iliac spines, sacrum, greater trochanter, midpoint of the femur, lateral and medial epicondyles of the femur, lateral and medial plateaus of the tibia, midpoint of the tibia, lateral and medial malleoli, the calcaneus, and the first and fifth metatarsal heads (Fig. 3). The sampling rate was set at 400 Hz. Kinematic and kinetic data were filtered using a zero-lag, fourth-order, Butterworth low-pass filter at a cutoff frequency of 10 Hz and 40 Hz, respectively, using MATLAB R2014a (MathWorks, Natick, MA, USA).

**Figure 4:**
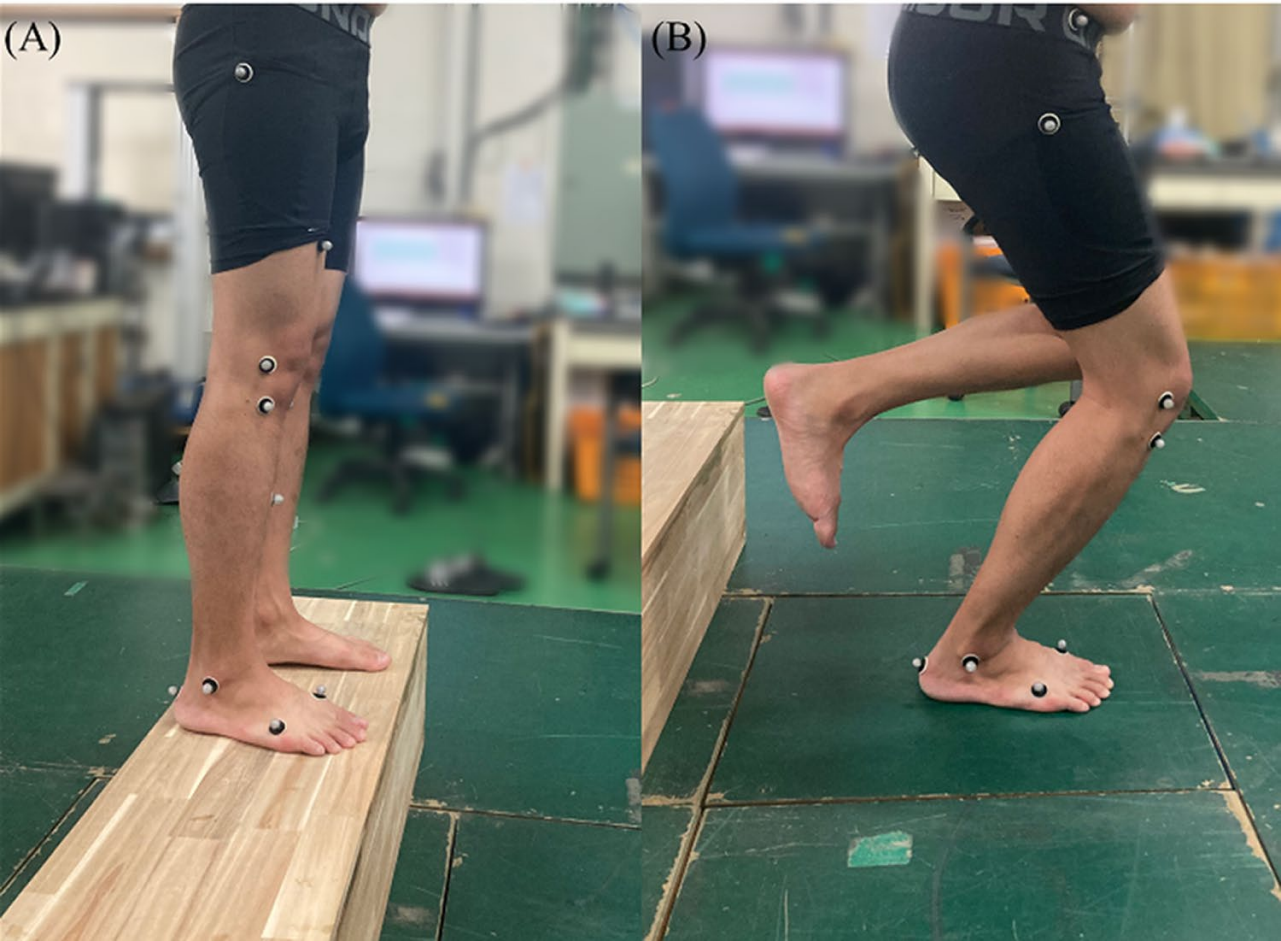
Experimental setup for drop single-leg landing test. (**A**) initial position of drop landing and (**B**) Final position of single-leg landing.

The participants executed a single-leg drop landing from a 0.3-m platform (Fig. 4). They were instructed to keep folding their arms across their chest (as much as possible without trunk movement to remove the effect of lateral trunk tilt on knee valgus moment^37^) and stepping off (without jumping up) with the dominant leg on the force plate. The dominant limb was defined as the preferred leg used to kick a ball. The participants were asked to “land as naturally as possible” and remain balanced for at least 2 s after landing. Before the actual trials, each participant was instructed to perform over 10 times practice trials to become familiar with the procedures and instrumentation. After practical trials, 5 min of rest was provided. A 1 min of rest period was given between each actual trial for phosphocreatine restoration^38^. To remove the effect of foot progression angle on knee valgus moment^39^, if the foot was not facing straight ahead, then the corresponding trials were discarded. A successful landing trial required each participant to step off the platform and adopt a stable landing posture without losing balance for at least 2 s after landing. Three successful trials were performed for each participant The drop vertical jump is more demanding task compared with drop landing due to the subsequent task after landing but the kinetic variable between drop landing and the drop vertical jump are similar at impact phase before the jump^40^. Thus, in this study, the participants perform the drop landing instead the drop vertical jump to control landing demand^41^.

The joint angle and angular velocities were calculated using the filtered 3D marker coordinate data using MATLAB. A right-handed, Cartesian-coordinate system was defined for the foot, shank, thigh, and pelvis segments to describe the 3-dimesional position and orientation of those segments. Global and segment axis systems were established with the x-axis designated as positive in the anterior direction from the participant, the y-axis positive to the left, and the z-axis positive in the upward direction^42^. Hip joint center was determined using trochanter markers and locating them at 25% of the inter-trochanteric distance^43^. The knee joint was determined using the midpoint of a line between the medial and lateral tibial plateau. The ankle joint center was determined using the midpoint of markers placed on the medial and lateral malleoli. The hip, knee and ankle joint angles were calculated using Euler angle rotations of the femur relative to the pelvis, of the tibia relative to femur and of the foot relative to tibia. All dynamic kinematic movements were calculated by subtracting the static posture values from the dynamic values^44^. Three‐dimensional intersegmental forces and external moments were calculated by submitting the filtered kinematic and GRF data to conventional inverse dynamics analysis using MATLAB. The kinematics and kinetics of the lower extremity joint were analyzed between the initial contact (over 20 N)^45^ and maximum knee flexion. All data were normalized to 100% of the landing phase. The GRF was normalized to body weight, and all joint moments were normalized to body weight and height.

An independent t-test was used to compare kinematics (initial contact ankle plantar flexion, ankle ROM in sagittal plane, knee flexion, and knee ROM in sagittal plane) and kinetics (peak vGRF, ankle plantarflexion, ankle inversion, knee extension, and knee valgus moment) between sex. Pearson correlation coefficients were measured to determines a relationship between the initial contact ankle plantar flexion, ankle ROM, and peak ankle plantar flexion moment with the peak knee valgus moment. The strength of correlation was interpreted as negligible (r < 0.29), low (0.30–0.49), moderate (0.50–0.69), high (0.70–0.89), and very high (0.90–1.00)^46^. Cohen’s d effect size wase calculated to determine the magnitude of the magnitude of the difference in the variable between sexes. Cohen’s d was classified as weak (d ≤ 0.2), small (d = 0.2–0.5), moderate (d = 0.5–0.8), or large (d ≥ 0.8)^47^. All statistical analyses were conducted using MATLAB or G*Power.

## Data Availability

The datasets generated during and/or analyzed during the current study are available from the corresponding author on reasonable request.
